# Four-year trends in the treatment of cerebral aneurysms in Poland in 2009-2012

**DOI:** 10.1007/s00701-014-2006-z

**Published:** 2014-02-06

**Authors:** Tomasz Tykocki, Kacper Kostyra, Marcin Czyż, Bogusław Kostkiewicz

**Affiliations:** 1 Department of Neurosurgery, Institute of Psychiatry and Neurology, Sobieskiego Street No 9, Warsaw, 02-957 Poland; 2 Department of Neurosurgery, Central Clinical Hospital Ministry of Interior in Warsaw, Warsaw, Poland; 3 Department of Neurosurgery, Wroclaw Medical University, Wroclaw, Poland

**Keywords:** Intracranial aneurysms, Clipping, Endovascular treatment, Trends

## Abstract

**Background:**

The dilemma concerning the appropriate treatment of the intracranial aneurysms (IAs) has not yet been resolved and still remains under fierce debate. This study refers to the recent trends in the use of and outcomes related to coiling compared with clipping for unruptured and ruptured IAs in Poland over a 4-year period.

**Methods:**

The analysis refers to treatment of IAs performed in Poland between 2009-2012. Patients’ records were cross-matched by ICD-9 codes for ruptured SAH (430) or unruptured cerebral aneurysm (437.3) along with codes for clipping (39.51) and coiling (39.79, 39.72, or 39.52). Multivariable logistic regression was used to compare in-hospital deaths, hospital length of stay (LOS), therapy allocation and aneurysm locations in unruptured vs. ruptured and clipped vs. coiled groups. Differences in the number of procedures between 16 administrative regions were standardized per 100,000 people.

**Results:**

In 2009-2012, 11,051 procedures were identified, including 5,968 ruptured and 5,083 unruptured aneurysms. Overall increase was 2.3 % in clipping and 13.1 % in coiling; a significant trend was found in endovascular procedures (p = 0.044). Ruptured aneurysms were clipped more frequently (OR = 1.66;); in unruptured IAs, endovascular procedure was preferred 3.5 times more than clipping. The annual in-hospital mortality was 7.6 % in clipping and 6.7 % in endovascular treatment. LOS was two times longer after clipping in unruptured aneurysms (OR = 2.013). After the procedures were standardized per 100,000 people, the average for Poland was established as 9.09 in 2009, 10.86 in 2010, 10.55 in 2011, and 11.49 in 2012. This index had the highest values in Mazovia (12.9, 2009; 15.4, 2010; 17.4, 2011; 18.6, 2012.

**Conclusions:**

Data analysis revealed an increase in overall number of IAs treated in Poland between 2009-2012. A significant upward trend of endovascular procedures was found, whereas the number of clipped aneurysms remained relatively steady over the study period.

## Introduction

The dilemma concerning the appropriate treatment of intracranial aneurysms (IAs) has not yet been resolved and still remains under fierce debate. The introduction of Guglielmi detachable coils in 1991 [9] initiated a new epoch in cerebrovascular therapy and opened the door for endovascular procedures in the management of IAs. Thus, the hegemony of surgical clipping was broken by aneurysm coiling causing shifting treatment paradigms. The number of endovascular procedures has grown steadily since 1990s [6, 10, 11, 14]. At the same time, the number of clipped IAs had a downward trend with a significant decrease in the group of unruptured aneurysms [1, 4, 6, 10]. A marked change in the management of IAs in favour of coiling has been noticed since the publication of the International Subarachnoid Aneurysm Trial (ISAT) in 2002 [17]; additionally, the total number of any intervention, including both clipping and coiling, has also radically increased. Interestingly, the last report from the Barrow Study provided some new data on this intriguing issue. At 3-year follow-up, patients in the clip group reached a significantly higher degree of aneurysm obliteration and a significantly lower rate of recurrence and retreatment, but embolization showed a more favourable outcomes [23].

The excellent and sophisticated scientific descriptions and trend analysis in the treatment of IAs predominantly come from U.S. data and at the same time there is a lack of similar reports from other regions.

In this study, the authors present 4-year trends in the treatment of IAs in Poland. Data includes endovascular and neurosurgical interventions, ruptured and unruptured IAs and takes into account the differences between 16 administrative regions of Poland.

## Methods

The present statistical analysis refers to IAs treated in Poland between 2009 and 2012. These procedures are grouped according to the system of diagnosis-related groups (DRG) (pol. Jednorodne Grupy Pacjentów, JGP), as proposed by the National Health Fund (pol. Narodowy Fundusz Zdrowia, NFZ). Data were obtained from the NFZ database and supplemented by the official correspondence with the NFZ.

Patients’ records were cross-matched by ICD-9 codes for ruptured SAH (430) or unruptured cerebral aneurysm (437.3) along with procedure codes for aneurysmal clipping (39.51) and coiling (39.79, 39.72, or 39.52). Additionally, all aneurysms were subgrouped into either the anterior or posterior cerebral circulation location. In the first stage, the absolute numbers of procedures were estimated, followed by the comparison of results between unruptured vs. ruptured, clipped vs. coiled and posterior vs. anterior cerebral circulation aneurysms. Demographic characteristic for the Polish population and differences in the number of procedures between 16 administrative regions (voivodships) were standardized per 100,000 people. The population data was obtained from Statistical Yearbooks of the Central Statistical Office. The two main cohorts (unruptured and ruptured aneurysms) were compared against one another for two primary end points—in-hospital death and hospital length of stay (LOS). Secondarily, similar comparison in the main cohorts for three other features, unruptured vs. ruptured, clipped vs. coiled and posterior vs. anterior cerebral circulation aneurysms, were conducted. Patients with both a ruptured and unruptured aneurysm in one hospitalization were included in the ruptured group. We excluded patients who were listed with procedure codes for both clipping and coiling, and those who received diagnosis codes for both an unruptured aneurysm and SAH.

### Statistical analysis

Multivariable logistic regression was used to calculate the odd ratios and 95 % CIs for the comparison of endovascular treatment and surgical clipping for the in-hospital mortality, sex, length of stay and the anatomical origin of the aneurysms. We also calculated and tabulated category-level compound annual growth rates (CAGRs) for the whole analyzed period. Trends over time were modeled using simple linear regression and tested for significance with analysis of variance (ANOVA), assuming that the dependent variables are numbers of procedures and time points (years) are independent variables. Statistical significance was defined as a type I error <0.05.

## Results

From 2009 to 2012, 11,051 procedures associated with the treatment of IAs were identified, including 5,968 ruptured and 5,083 unruptured aneurysms.

The mean age was 52.3 ± 3.8 years and there were 6,666 women (60.3 %). Five thousand one hundred and twenty-five (46.4 %) aneurysms were treated by neurosurgical clipping and 5,926 (53.6 %) by endovascular procedures. The overall in-hospital mortality rate was 7.1 % and the mean length of stay 9.3 ± 0.9 days. The location ratio of anterior to posterior circulation aneurysms was 6:1 (9,228:1,545); the highest number of IAs was in the ICA 3,769 (34 %) and ACA 3,199 (29 %) territory (Table 1).

**Table 1:** Comparison of the selected factors between clipping and endovascular groups

	Endovascular N = 5,926	Clipping N = 5,125	Odds ratio*	CI 95 %
In-hospital mortality	396 (6,7 %)	391 (7,6 %)	1.158	1.001 to 1.339
Age (years)	54.9 ± 4.3	51.3 ± 3.5	0.910	0.867 to 1.012
No. of women	3,827 (64.6 %)	2,839 (55.4 %)	0.685	0.634 to 0.739
LOS (days)	6.6 ± 0.8	11.9 ± 0.7	2.049	1.274 to 3.296
Posterior circulation (n)	1,205 (20.3 %)	350 (6.8 %)	0.294	0.260 to 0.334
ICA (n)#	2,092 (35.3 %)	1,675 (32.7 %)	0.886	0.818 to 0.959
MCA (n) #	726 (12.2 %)	1,534 (29.9 %)	3.054	2.768 to 3.370
ACA (n) #	1,903 (32.1 %)	1,566 (30.6 %)	0.922	0.850 to 0.999

Pooled analysis for 2009-2012

LOS – length of stay

*odds ratio adjusted per year

# territory of internal carotid artery (ICA); middle cerebral artery (MCA); anterior cerebral artery (ACA)

### Comparison of the surgical clipping and endovascular treatment

**Fig. 1:**
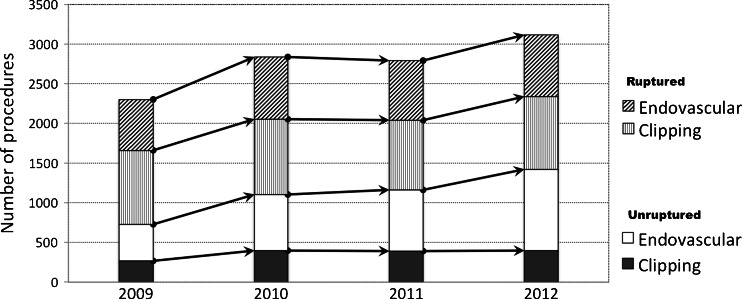
Column graph bar presenting number of procedures in 2009-2012 for ruptured and unruptured aneurysms

**Fig. 2:**
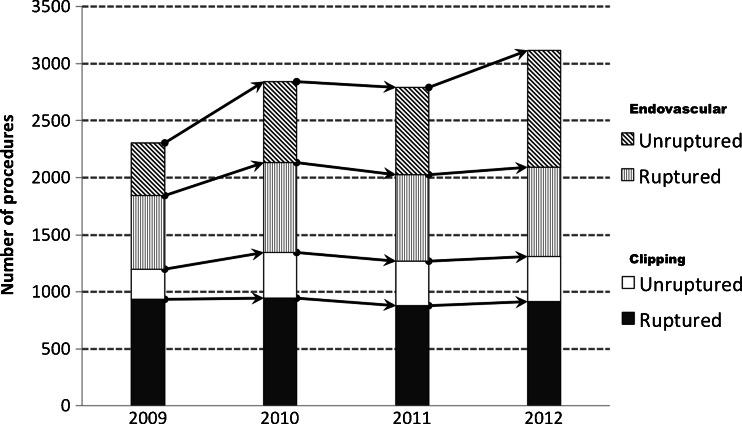
Column graph bar presenting number of aneurysms treated by clipping and endovascular therapy in 2009-2012

An overall increase in the number of all IAs treated between 2009-2012 was observed, the CAGR was calculated as 6 % for the entire period, but the change in volume was not significant by ANOVA (p = 0.112) (Table 2). An increase in the number of procedures for neurosurgical clipping by 2.3 % and endovascular treatment by 13.1 % was observed; however, a significant trend was found only in the endovascular group (p = 0.044). There has been a steady increase in the proportion of unruptured aneurysms including both procedures between 2008 and 2009 (CAGR, 18.2 %; p = 0.036); a similar increment was found for the endovascular group (CAGR, 22.1 %; p = 0.023), but not in the clipping one (Fig. 1). The overall number of clipped aneurysms remained relatively steady over the entire period, but the volume of unruptured aneurysms showed an upward trend (Fig. 2). Posterior circulation aneurysms have increased almost three times in the study period with a significant increment between 2009 and 2010; this tendency was associated with a remarkable change in the treatment choice of these IAs favouring endovascular treatment. The multivariate pooled analysis for the entire study period adjusted per year showed that the average length of hospital stay was longer in endovascular treatment (OR = 2.049; 95 % CI 1.274 to 3.296). Posterior circulation aneurysms were 3.4 times more likely to be coiled than clipped; the opposite relationship was found for MCA aneurysms being more frequently clipped than coiled (OR = 3.053; 95 % CI 2.768 to 3.370). The annual in-hospital mortality was estimated as 7.6 % in clipping and 6.7 % in endovascular treatment; the difference did not reach statistical significance. Annual comparison between clipping and endovascular treatment was presented in Table 3.

**Table 2:** Number of aneurysm treatment procedures in Poland, **2009-2012**

	Calendar year					Compound annual growth rate (%)
	2009	2010	2011	2012	p*	2009-2012
Clipping + endovascular	3,501	4,185	4,063	4,427	0.112	6.0 %
unruptured	728	1,108	1,162	1,421	0.036	18.2 %
ruptured	2,773	3,077	2,901	3,006	0.400	2.0 %
posterior circulation	166	396	412	495	0.081	31.4 %
Clipping	1,199	1,345	1,269	1,312	0.461	2.3 %
unruptured	267	399	393	397	0.233	10.4 %
ruptured	932	946	876	915	0.484	-0.5 %
posterior circulation	53	53	79	79	0.104	10.4 %
Endovascular	1,103	1,495	1,525	1,803	0.044	13.1 %
unruptured	461	709	769	1,024	0.023	22.1 %
ruptured	642	786	756	779	0.266	5.0 %
posterior circulation	113	343	333	416	0.112	38.5 %

* P value is the result of ANOVA F test for significance of a linear trend over time

**Table 3:** Annual comparison of the selected factors in clipping and endovascular groups

	2009		2010		2011		2012	
	Clipping n = 1,199	Endovascular n = 1,103	Clipping n = 1,345	Endovascular n = 1,495	Clipping n = 1,269	Endovascular n = 1,525	Clipping n = 1,312	Endovascular n = 1,803
In-hospital mortality	92 (7.7 %)	67 (6.1 %)	111 (8.3 %)	94 (6.3)	91 (7.2 %)	102 (6.7 %)	96 (7.3 %)	133 (7.4 %)
Age (years)	55.3 ± 3.3	52.4 ± 4.1	54.6 ± 2.9	49.1 ± 3.1	53.1 ± 4.6	52.8 ± 4.6	56.8 ± 3.7	51 ± 4.8
No. of women	647 (54 %)	699 (63.4 %)	732 (54.4 %)	963 (64.4 %)	714 (56.3 %)	990 (64.9 %)	746 (56.9 %)	1,175 (65.2 %)
LOS (days)	11.3 ± 1.1	7.2 ± 0.9	12.8 ± 0.9	7.1 ± 0.8	11.5 ± 1.2	6.7 ± 0.6	12.1 ± 0,8	5.5 ± 0.7
ICA (n)#	381 (31.8 %)	424 (38.4 %)	457 (34 %)	531 (35.5 %)	412 (32.5 %)	526 (34.5 %)	425 (32.4 %)	611 (33.9 %)
MCA (n) #	372 (31 %)	139 (12.6 %)	405 (30.1 %)	172 (11.5 %)	370 (29.2 %)	191 (12.5 %)	387 (29.5 %)	224 (12.4 %)
ACA (n) #	366 (30.5 %)	427 (38.7 %)	417 (31 %)	449 (30 %)	389 (30.7 %)	475 (31.1 %)	394 (30 %)	552 (30.6 %)

LOS – length of stay

# territory of internal carotid artery (ICA); middle cerebral artery (MCA); anterior cerebral artery (ACA)

**Fig. 3:**
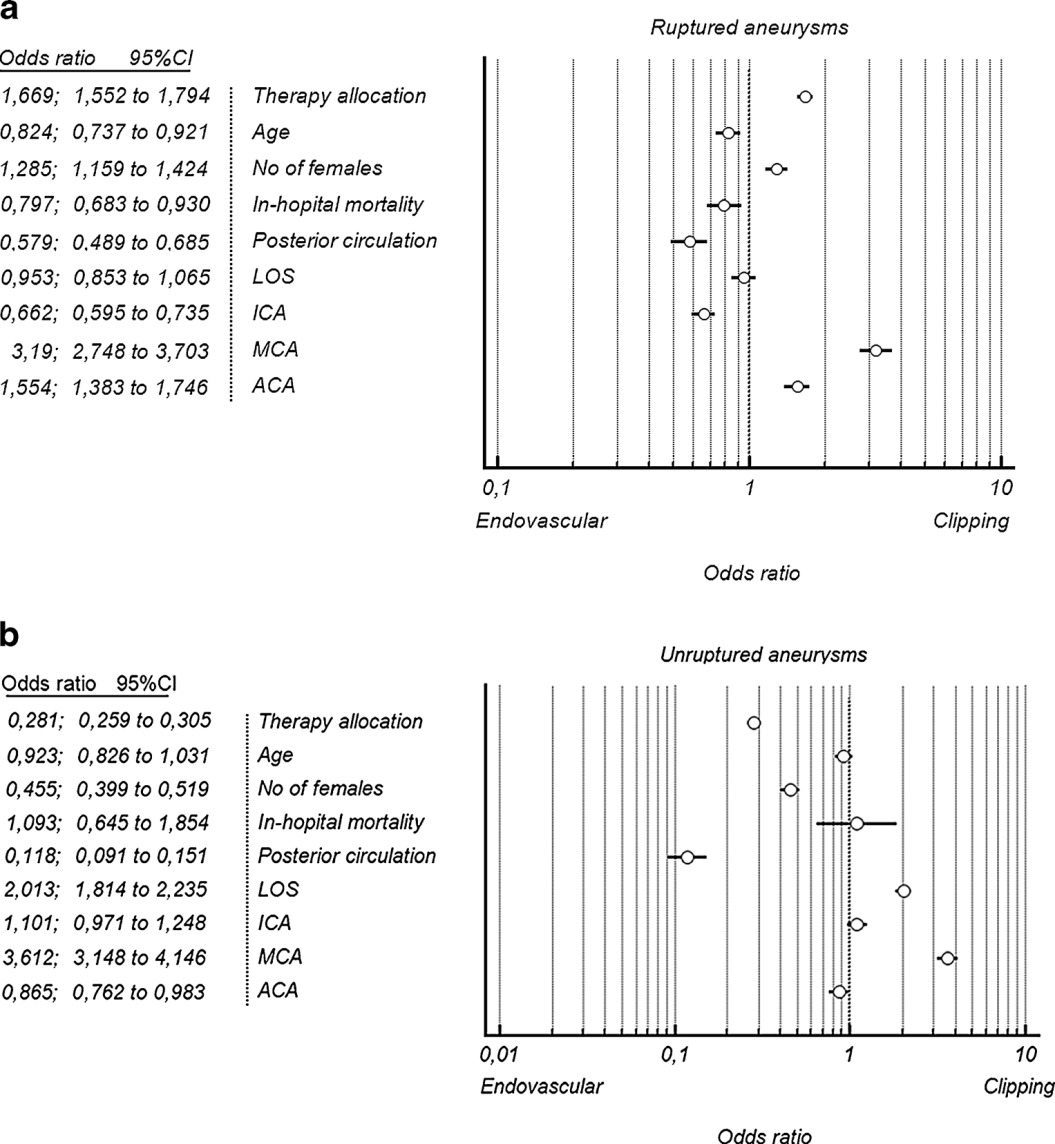
Forest plots comparing selective factors between endovascular treament and clipping in unruptured (**a**) and ruptured aneurysms (**b**)

The differences between these two groups were more pronounced when comparing ruptured and unruptured aneurysms (Fig. 3a, b). Ruptured aneurysms were clipped more frequently (OR = 1.66; 95 % CI 1.552 to 1.794), and in the unruptured group endovascular procedure was preferred 3.5 times more than clipping. The length of hospital stay was almost equal after SAH, whereas in unruptured aneurysms, the hospital stay was two times longer after clipping (OR = 2.013; 95 % CI 1.814 to 2.235). Posterior circulation aneurysms were more likely to be coiled in both ruptured (OR = 1.7; 95 % CI 1.45 to 2.04) and unruptured (OR = 8.48; 95 % CI 6.62 to 10.99) groups. On the other hand, MCA aneurysms were allocated mostly for clipping (OR = 3.19; 95 % CI 2.748 to 3.703 in the SAH group; OR = 3.612; 95 % CI 3.148 to 4.146 in the non-SAH group).

### Geographical variations in the treatment of cerebral aneurysms

In half of the 16 administrative regions, an increase in the number of clipped and coiled aneurysms over the study period was found (Table 4). The highest CAGR was 14 % for clipped aneurysms over the study period in Greater Poland; the change was from 34 in 2009 to 58 in 2012. The greatest progression in endovascular treatment was found in the Lublin region with a CAGR of 33.3 % (62 in 2009 to 196 in 2012). The cumulative number of procedures in the 4-year period was the highest in Mazoivia and reached 1,680 (average per year – 420/78.59) in clipping and 1,710 (average per year – 427.5/55.51) in endovascular treatment. There was a reduction in the number of clipped aneurysms in three regions between 2009-2012 (Lesser Poland -1 %; Swietokrzyski Region -11 %; Opole Region -8 %). No endovascular treatment was found in one region (Lubusz Region) throughout the entire study period. In two regions there was a downward tendency in the number of endovascular procedures (Opole Region -6.9 %; Podlasie Region -8 %).

**Table 4:** Number of aneurysm treatment procedures in sixteen Polish regions in clipping and endovascular groups

	2009		2010		2011		2012		Compound annual growth rate (%); 2009-2012	
Region/Voivodship	clipping	endovascular	clipping	endovascular	clipping	endovascular	clipping	endovascular	clipping	endovascular
Lower Silesian	38	145	73	157	63	215	41	242	2 %	14 %
Kuyavian-Pomeranian	54	114	96	116	104	119	56	149	1 %	6.9 %
Lublin Region	119	62	155	148	135	160	129	196	2 %	33.3 %
Lubusz Region	38	0	40	0	45	0	43	0	3 %	0 %
Lodz Region	73	55	91	84	85	48	77	65	1 %	4.3 %
Lesser Poland	133	47	155	78	140	82	126	95	-1 %	19.2 %
Mazovia	312	367	416	397	459	457	493	489	12 %	7 %
Opole Region	34	8	21	9	21	7	24	6	-8 %	-6.9 %
Podkarpacie Region	18	0	19	50	22	93	23	42	6 %	0.0 %
Podlasie Region	23	58	27	50	25	83	33	41	9 %	-8 %
Pomeranian	68	0	87	4	105	35	105	48	11 %	0.0 %
Silesian	113	141	160	170	135	236	151	221	8 %	11.9 %
Swietokrzyski Region	35	22	33	52	26	56	22	80	-11 %	38 %
Warmian-Mazurian	35	0	28	10	34	31	33	15	-1 %	0.0 %
Greater Poland	34	221	66	378	58	477	58	557	14 %	26.0 %
West Pomeranian	84	66	88	78	100	86	92	122	2 %	17 %

**Fig. 4:**
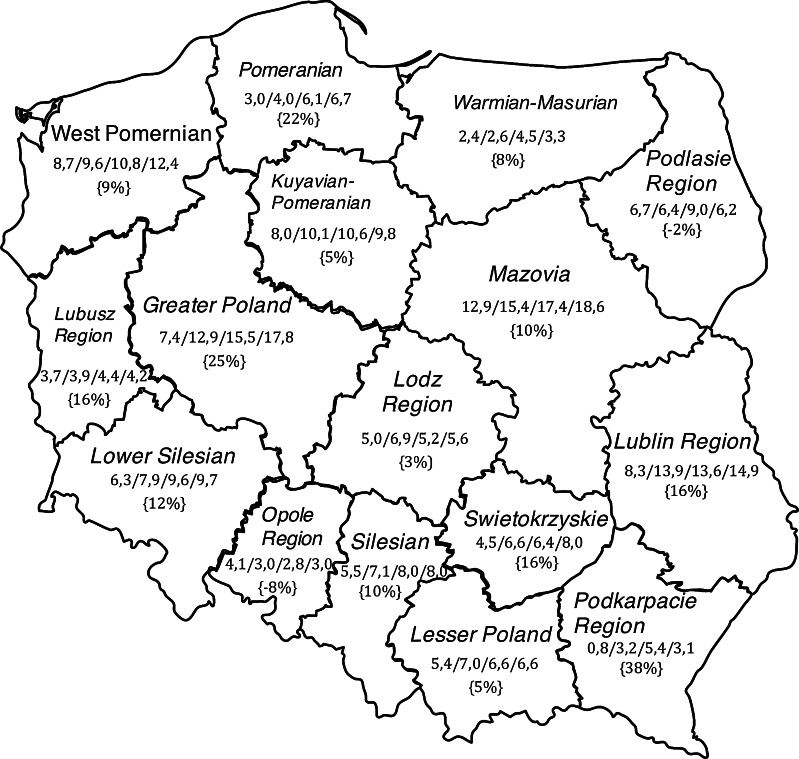
Administrative map of Poland showing the annual number of overall aneurysm treated in 2009-2012 in 16 regions. Compound annual growth rates (%) between 2009 and 2012 are presented in parentheses

After the procedures were standardized per 100,000 people, the average for Poland was established as 9.09 in 2009; 10.86 in 2010, 10.55 in 2011; 11.49 in 2012. This index had the highest values in Mazovia (12.9 in 2009; 15.4 in 2010; 17.4 in 2011; 18.6 in 2012) with CAGR of 10 %. In three other regions the index (procedures/100,000) was above the average for Poland (Greater Poland, Lublin Region, West Pomeranian) (Fig. 4).

## Discussion

In this retrospective analysis, data from the Polish national database was used to investigate trends in aneurysm treatment patterns in Poland for the 4-year interval. A moderate increase in the overall number of aneurysms treatment, both by clipping or endovascular treatment, was noted over the study period. This increase is represented predominantly by unruptured and posterior circulation aneurysms with yearly increase by 18.2 % and 31.4 %, respectively. For both ruptured and unruptured aneurysms, the trend was toward an increased use of endovascular procedures by 13.1 % annually. The relative increase in coiling usage was greater in patients with no SAH than in those with ruptured aneurysms. Generally, clipping had a slight upward tendency, while the number of clipped ruptured aneurysms decreased marginally. A significant growing trend was found in the number of unruptured aneurysms and endovascular procedures over the 4-year study period. A marked shift in the therapy allocation from surgical clipping to endovascular coiling was noted for both ruptured and unruptured aneurysms after publication of the results of the International Subarachnoid Aneurysm Trial (ISAT) in 2002 [17]. There was a prominent change in the number of procedures performed prior to and after the ISAT study; namely, there was an increase in endovascular procedures in the post-ISAT period (3 % vs. 17 %), a decrease in surgical procedures (31 % vs. 23 %), and a nonsignificant increase in proportion of patients who get any treatment (surgical or endovascular) [20]. A European study from the United Kingdom reported that the proportion of patients undergoing surgical treatment decreased from 51 % to 31 % while endovascular treatment of aneurysms increased from 35 % to 68 % since publication of ISAT results [8]. Based on US data, endovascular approaches increased in utilization from 11 to 43 % of unruptured and 5 to 31 % ruptured IAs between 1998-2003 [1]. The proportion of patients treated endovascularly increased from 49 % before to 87 % prior and after the ISAT [15]; in France over 85 % of patients with aneurysms underwent coil embolization event before the ISAT trial began [5].

The legitimate factors related to the shift in treatment selection include subsequent technologic advances such as more prevalent usage of stent-assistant coiling, increased availability of endovascular procedures. There have also been substantial improvements in endovascular technology, such as bioactive coils, expandable coils, and complex-shaped coils during the same period, which may account for some of observed increase in utilization [7, 16].

Piotin et al. concluded that stent-assisted coiling reduced the recurrence rate, but in exchange, a higher rate of morbidity and mortality has appeared. The overall rate of permanent, procedure-related morbidity and mortality was 12 % for the stent-treated cohort compared with 5 % in the non-stent-treated group [18].

Based on the data from this study, clipping was three times more often utilized than coiling for treatment of MCA IAs. There is a disparity across Europe in the treatment selection for MCA IAs. Northern European countries strongly favour coiling in comparison with the rest of Europe, where clipping is the treatment of choice [3]. The rationale is based on excellent results in surgical series with MCA IAs [21, 25]. The main arguments against endovascular treatment include broad necks, branches frequently originate from the base or side wall of the aneurysm, increasing the risk of branch occlusion with coiling, large or giant size, intraluminal thrombus, fusiform or complex morphology [21]. However, the recommendations for MCA IAs are based not on high-powered studies but only on personal experience. Endovascular treatment may thus be proposed as an alternative to surgical clipping with very promising results at this location [2, 12, 19]. Contrary to MCA IAs, those in posterior circulation were over three times more likely to be coiled than clipped. This difference was even more scattered for unruptured IAs favouring endovascular treatment 8.5 times more often than the surgery. The tendency to coil posterior circulation IAs is mostly associated with the more complex and challenging operative approach and, what is more, extensive experience in neurovascular surgery. The dominance of endovascular treatment in the field of vertebrobasilar aneurysms is obvious across the whole of Europe [3]. ISAT’s impact in the US has been weaker, but in a series of 100 consecutive patients that had been treated by one neurosurgeon using either surgical clip application or endovascular coil embolization, none of the 17 posterior circulation aneurysms were clipped [13]. Literature review by Sanai et al. found comparable overall outcomes after clipping (n = 2,377) or coiling (n = 857); however, endovascular therapy was associated with higher incomplete aneurysm occlusion (8.2 % vs. 4 %), recurrence (9.9 % vs. 0 %), rehemorrhage (5 % vs. 0.2 %) [22]. In the BRAT study, the outcomes of posterior circulation aneurysms were significantly better in the coil group than in the clip group after the first year of follow-up, and this difference persisted after 3 years of follow-up [23]. However, the perception that endovascular therapy is superior to microsurgical clipping for posterior circulation aneurysms is not based on clear evidence.

This study showed that the annual in-hospital mortality was 1.2 times higher in the clipping group (7.6 %) compared to endovascular treatment (6.7 %). Endovascular coiling was associated with slightly lower in-hospital mortality for patients with unruptured factors, but higher in SAH group in comparison to clipping. Including all studied IAs, surgical clipping had two times longer mean length of hospitalization than the endovascular treatment (11.9 ± 0.7 vs. 6.6 ± 0.8 days). Similar finding was noted in unruptured group (OR = 2.013), whereas after SAH LOS was almost equal in both methods of treatment. Data from the Nationwide Inpatient Sample showed that in-hospital mortality decreased from 9.7 % to 6.9 % in the clipped group and from 7.2 % to 6.7 % in the endovascular group over a decade (1993-2003) [1]. Cowan et al. reported that in unruptured IAs endovascularly treated patients had a shorter LOS (2 compared with 5 days) but the LOS in SAH patients was shorter in clipped aneurysms (15 compared with 17 days median) [6]. Similar data were shown by Hoh et al. in the 2-year retrospective study of 545 patients [10]. The longer LOS and higher in hospital mortality after sole endovascular procedures may result from the lack of decompressive craniectomy in severe brain edema with mass effect, brainstem compression and persistent intracerebral hematoma in some cases after SAH [7].

However, early after endovascular therapy had been introduced, it was used mainly in older patients, poor grade subarachnoid hemorrhage, and large or giant aneurysms. Although the proportions have recently changed toward being more balanced, a slight skew to shift more severe cases to coiling still exists [7].

There is a high disparity across the geographical regions in Poland in the number of procedures. This is consistent with a previous study referring to the overall number of neurosurgical procedures, in which a significant regional scatter of neurosurgical operations was found [24]. Regional unequal ratio in the number of clipped to coiled IAs reflects the uneven development of the endovascular therapy, especially when in some regions a total lack or only a few endovascular procedures could be found. Bradac et al. in the European internet aneurysm study found that Eastern European countries use endovascular coiling much less than the rest of Europe [3]. However, the global number of clipped and coiled IAs was comparable in our study, with the predominance of endovascular treatment (5,926 vs. 5,125) over the entire period.

## Conclusions

The trend analysis revealed an increase in overall number of aneurysms treated between 2009-2012. A significant upward trend of endovascular procedures was found, whereas the number of clipped aneurysms remained relatively steady over the study period. Middle cerebral artery aneurysms were predominantly allocated to surgical clipping, whereas posterior circulation aneurysms were mostly treated by endovascular therapy in both unruptured and ruptured groups. The length of hospital stay was almost equal after SAH, but in unruptured aneurysms the hospital stay was two times longer after clipping.

A high disparity in the number of procedures across the geographical regions in Poland was pronounced; the difference was especially noticeable in endovascular therapy.
